# Ethical challenges posed by clinical trials in preterm labor: a case study

**DOI:** 10.1186/s12978-017-0427-x

**Published:** 2017-12-14

**Authors:** Sofía P. Salas

**Affiliations:** Program of Ethics and Public Policies in Human Reproduction, Faculty of Medicine, Universidad Diego Portales, Santiago, Chile

**Keywords:** Research ethics, Pregnant women, Informed consent, Preterm labor, Tocolysis

## Abstract

This paper explores the ethical implications of a randomized double-blind clinical trial aimed to determine effectiveness and safety of an oxytocin receptor antagonist versus a betamimetic in the treatment of preterm labor, presented to a teaching hospital affiliated with a private university in Santiago, Chile. Though this trial protocol fulfills one of the conditions under which pregnant women could be enrolled in a clinical trial—the intervention has the potential to benefit the pregnant woman (by reducing adverse effects associated to salbutamol administration) and her fetus (if the new drug prolongs pregnancy)—there are some specific ethical issues raised. First, when to obtain consent is an important issue for clinical trials involving acute and unforeseen conditions that affect pregnant woman, e.g. preterm labor. Second, research must address the risk/benefit ratio for these two interdependent individuals, providing a good prospect of low risk and adequate benefit for both of them. Thirdly, specifically when a study is sponsored by a high-income country and conducted in a low- or middle-income country, decisions regarding ancillary care provisions for research participants should be made in advance. Lastly, researchers must consider the requirements for paternal consent based on cultural contexts.

## Background

Preterm birth, defined as birth occurring between 20 and 36 weeks of gestation, is a major cause of perinatal morbidity and mortality. Preterm complications are the leading cause of death among children under the age of five, and in low-income settings, nearly half of births occurring before 32 weeks of gestation result in death due to lack of cost-effective care [1, 2]. The acute use of a tocolytic drug to prolong pregnancy for up to 48 h can be useful in order to provide a window for administration of antenatal corticosteroid or in utero fetal transfer to an appropriate neonatal healthcare setting.

A study design aimed to compare effectiveness and safety of the oxytocin receptor antagonist (atosiban) versus a betamimetic (salbutamol) in the treatment of preterm labor was presented at the institutional review board (IRB) of a teaching hospital affiliated with a private university in Santiago, Chile. In 1999, when this study protocol was submitted, there was no clear evidence on which tocolytic drug was preferable. Although the most frequently used tocolytic agents were beta-adrenergic agonists (betamimetics), maternal side effects—cardiovascular adverse events are reported in nearly 80% of the women—usually caused early discontinuation of the therapy despite its effectiveness in delaying birth for more than 48 h [1, 3, 4].

To blind the study treatment, a double-dummy technique was used (the study medications were identical in shape, size, and color). Inclusion criteria included maternal age between 16 and 44 years, intact membranes, between 24 weeks’ and 34 weeks’ gestation. Reported or documented uterine activity, and cervical dilation between 2 cm and 4 cm in an otherwise normal singleton pregnancy. The primary outcome was preterm birth (<37 weeks); secondary outcomes were preterm birth within 48 h of randomization, at least two doses of corticosteroid administered prior to delivery, and preterm birth within seven days of randomization. Both groups received standard obstetric and neonatal care; there were no other interventions associated with their participation in the trial other than strict data registration and non-invasive neonatal follow-up.

### Ethical discussion

A recent Committee Opinion of the American College of Obstetricians and Gynecologists regarding the inclusion of women as research participants recommends that pregnant women in research trials should be defined as “scientifically complex”—rather than “vulnerable”—because they are able to protect their own interests and give informed consent [5]. Accordingly, present debates have moved from justifying the inclusion of pregnant women in clinical trials to justifying their exclusion in what has been called the “second wave” [6]. A critical question is how to balance potential risks to the fetus with respect to benefits to the pregnant woman, particularly if she is enrolled in life-saving trials that could provide evidence based effective treatment [7, 8]. Pregnant women do require special protections as research participants due to a legitimate concern about the protection of both the woman and her fetus [9, 10]. As stated in the revised *International Ethics Guidelines for Health-related Research Involving Humans* of the Council for International Organizations of Medical Sciences (CIOMS), there are two conditions under which pregnant women can be enrolled in a clinical trial: 1. when the interventions or procedures have the potential to benefit either the pregnant woman or her fetus, in which case risks must be minimized and outweighed by the prospect of potential individual benefit, or 2. when the research has no potential direct benefits for the pregnant women, the risks must be minimized and no more than minimal, and the purpose of the research must be to obtain knowledge relevant to the particular health needs of the pregnant women or their fetuses [10]. In this case study, the intervention was necessary to treat a condition that is only present in pregnant women with premature labor and has the potential to benefit both the pregnant women (by reducing the side effects associated with use of betamimetics) and their fetuses (by delaying birth). At the time the study was presented to the local IRB, there was no legal framework related to research ethics in Chile; the law approved in 2006 regarding human scientific research does not describe specific research considerations for any sub-population, except for a ban on embryo research [11]. In this paper, we focus on the specific ethical issues raised by clinical trials conducted with pregnant women experiencing preterm labor.

#### Timing to obtain consent

Preterm labor is an unforeseen and acute complication that threatens neonatal survival. Pregnant women with premature uterine contractions are usually anxious and in pain, and may not be in the best condition to participate in a full consent procedure, particularly when there is limited time for decision-making [12, 13]. The local IRB suggested that the maternity ward enrolling women with the condition should implement a pre-consent procedure during usual pregnancy checkups. This allowed enough time for women to consider enrollment—before the preterm labor condition was present—with a more comprehensive understanding of the known maternal risks and potential benefits for the newborn of using the standard treatment or the new drug. A woman’s refusal was documented in her medical records and her decision was respected. Only those women that had initially agreed to be enrolled were contacted for an abbreviated consent procedure if they started experiencing premature contractions. This strategy proved to be useful and did not limit recruitment. A similar approach of providing information about the study during routine pregnancy checkups to all potential participants has been implemented for other studies [14, 15]. Interestingly, a recent study that evaluated women’s perspectives regarding the informed consent process in acute peripartum conditions demonstrated that women preferred trial information to be provided during the antenatal period, not at the moment the acute condition was present [13].

#### Balance between the interests of the pregnant woman and the infant

This clinical trial is a good example of maternal-fetal conflict, since the use of betamimetics for treatment of premature labor poses important health risks for the pregnant woman that are generally accepted in exchange for gain in neonatal survival [16]. Therefore, it is important to address the risk/benefit ratio for these two interdependent individuals—a new tocolytic drug aimed to reduce maternal side effects should be equally effective in prolonging pregnancy for benefits in neonatal survival. In evaluating this trial, the local IRB considered that there was enough pre-clinical evidence that the new drug, atosiban, had fewer maternal side effects due to its lack of cardiovascular effects, and that it could likely delay labor due to its mechanisms of action as an oxytocin receptor antagonist [4]. In addition, the protocol included the administration of alternative tocolysis in the event of treatment failure according to local clinical practice. In current practice, atosiban is one of the two first-line tocolytic drugs, whereas betamimetics have been abandoned due to side-effects [17].

#### Ancillary care obligations

As has been expressed elsewhere, ancillary care received by research participants is an important issue, particularly when the study takes place in low-and middle-income countries [18]. If the sponsor or the medical researcher does not provide this care, the research participants may not have their health needs met. However, the extent of these obligations is not always obvious. In this particular trial, it was difficult to determine the duties of the sponsor towards the pregnant woman and the child in the event a premature delivery occurs, and what, if any, ancillary care should be provided. The local IRB considered that the study could be done safely in the teaching hospital, which already provided the best standard of care for premature newborns (i.e. access to intensive care unit, antenatal corticoid administration, and surfactant use). In this case, the costs of these treatments were covered by the corresponding social security, differentiating them from the costs of treatment for adverse events regarding the participation in the protocol. The sponsor should pay for any maternal complications secondary to cardiovascular adverse effects or for fetal or newborn complications associated with the experimental drugs that were used, but not for the women’s hospital bill or the intensive care unit for the premature baby in the event a premature delivery occurred. The rationale for this distinction was that proper neonatal care was already provided, so the health needs of the participants were met through standard care. However, in a different clinical scenario, e.g. a low-resource setting without a proper neonatal intensive care unit, the sponsors should provide this ancillary care, defined as the “healthcare that research participants need but that is not necessary to ensure the safety of scientific validity of the research…” [19, 20].

#### Paternal consent requirements

CIOMS guidelines argue that the requirement of individual informed consent by the pregnant woman is mandatory and that in no case should the permission of another person (spouse or partner) replace the woman’s consent [10]. Though local adaptations to include the authorization of the fetus’ father might be necessary in certain cultural contexts, the refusal of the woman to participate should prevail.

## Conclusions

Inclusion of pregnant women as research participants for acute and unforeseen medical conditions, such as threatened premature labor, raises important ethical questions that should be carefully analyzed by the local IRB. First, it is important to implement a pre-consent process during antenatal visits that will allow pregnant women to make an informed decision before the moment the acute condition is present. Second, the IRB should carefully evaluate the risk/benefit ratio for both the pregnant woman and her fetus. Third, it is important to consider what ancillary care that the sponsor should provide. Finally, depending on the prevailing local culture, the IRB should evaluate if the partner’s consent should be obtained in addition to the pregnant woman’s consent.

## Abbreviations

CIOMS: Council for International Organizations of Medical Sciences
IRB: Institutional review board
